# A specialized post-anaesthetic care unit improves fast-track management in cardiac surgery: a prospective randomized trial

**DOI:** 10.1186/s13054-014-0468-2

**Published:** 2014-08-15

**Authors:** Stefan Probst, Christof Cech, Dirk Haentschel, Markus Scholz, Joerg Ender

**Affiliations:** Department of Anaesthesiology and Intensive Care Medicine II, Leipzig Heart Centre, University of Leipzig, Struempellstrasse 39, Leipzig, 04289 Germany; Department of Anaesthesiology and Intensive Care Medicine, University of Leipzig, Medical Faculty, Liebigstrasse 20, Leipzig, 04103 Germany; Department of Anaesthesiology and Intensive Care Medicine, Heart Centre Coswig, Lerchenfeld 1, Coswig, 06869 Germany; Institute of Medical Informatics, Statistics, and Epidemiology, University of Leipzig, Härtelstraße 16-18, Leipzig, 04107 Germany

## Abstract

**Introduction:**

Fast-track treatment in cardiac surgery has become the global standard of care. We compared the efficacy and safety of a specialised post-anaesthetic care unit (PACU) to a conventional intensive care unit (ICU) in achieving defined fast-track end points in adult patients after elective cardiac surgery.

**Methods:**

In a prospective, single-blinded, randomized study, 200 adult patients undergoing elective cardiac surgery (coronary artery bypass graft (CABG), valve surgery or combined CABG and valve surgery), were selected to receive their postoperative treatment either in the ICU (n = 100), or in the PACU (n = 100). Patients who, at the time of surgery, were in cardiogenic shock, required renal dialysis, or had an additive EuroSCORE of more than 10 were excluded from the study. The primary end points were: time to extubation (ET), and length of stay in the PACU or ICU (PACU/ICU LOS respectively). Secondary end points analysed were the incidences of: surgical re-exploration, development of haemothorax, new-onset cardiac arrhythmia, low cardiac output syndrome, need for cardiopulmonary resuscitation, stroke, acute renal failure, and death.

**Results:**

Median time to extubation was 90 [50; 140] min in the PACU vs. 478 [305; 643] min in the ICU group (*P* <0.001). Median length of stay in the PACU was 3.3 [2.7; 4.0] hours vs. 17.9 [10.3; 24.9] hours in the ICU (*P* <0.001). Of the adverse events examined, only the incidence of new-onset cardiac arrhythmia (25 in PACU vs. 41 in ICU, P = 0.02) was statistically different between groups.

**Conclusions:**

Treatment in a specialised PACU rather than an ICU, after elective cardiac surgery leads to earlier extubation and quicker discharge to a step-down unit, without compromising patient safety.

**Trial registration:**

ISRCTN71768341. Registered 11 March 2014.

## Introduction

Anaesthesia for cardiac surgery has traditionally been provided with high-dose opioids and long-acting muscle relaxants, in the belief this technique was associated with optimal haemodynamic stability. The resulting prolonged postoperative ventilation and intensive care unit (ICU) length of stay (LOS) were considered acceptable compromises. Rising costs and the need for faster ICU turnover due to increased demand and reduced resources led to reducing the length of ICU stay after cardiac surgery [1,2]. Since the mid-1990s, intensified postoperative rehabilitation has established itself as the optimal approach to patient recovery. Fast-track treatment has become a popular and accepted standard because it allows for early extubation within six hours and consequently reduced LOS in the ICU and hospital [3-5]. A significant reduction in time to extubation (ET) without compromising patient's safety has been demonstrated in numerous studies [5-11]. Zhu *et al*. described in *Cochrane Database Systematic Review* a mean reduction of 5.99 hours (2.99 to 8.99 hours) due to implementation of a time-directed extubation protocol without increasing the risk of postoperative complications compared to standard care. Low-dose opioid anaesthesia will reduce mean ET by 7.40 hours (10.51 to 4.29 hours) compared to high-dose opioid anaesthesia [11].

Implementation of a dedicated fast-track protocol that allows not only for earlier extubation but also for earlier transfer from the ICU or post-anaesthetic care unit (PACU) to a step-down unit has been shown to be very effective in reducing ICU-LOS and the total length of hospital stay in retrospective studies [5,6,8]. Zhu *et al*. showed in a review that low-dose opioid anaesthesia was associated with 3.7 hours (−6.98 to −0.41) lower ICU LOS. Time-directed extubation protocols had 5.15 hours (−8.71 to −1.59) shorter length of stay in the ICU (0.4 to 8.7 hours) compared to conventional groups, as Zhu *et al*. described, although LOS in hospital was similar in both groups [11].

Utilised in combination, this approach has been associated with both significant cost savings, and also increased ICU bed capacity [12]. Most fast-track treatment protocols for cardiac surgery patients to date, however, have been implemented within the conventional ICU setting.

In general, it is possible to perform an extubation in the operating room (OR) with selected patient groups (OPCAB, MIDCAB and so on). This could make sense if no postoperative care unit is available or the fast-track concept is not continued at the ICU. There is still an ongoing discussion about the advantage of an early extubation in the OR. Straka *et al*. and Montes *et al*. were not able to show a reduced ICU LOS in cardiac surgery patients who get extubated in the OR [13,14]. Chamchad *et al*. found in a non-randomized observational study shorter ICU and hospital LOS. With an average ICU LOS of 27 hours, this study showed no additional benefit compared to early extubation in a PACU/ICU [15].

Nicholson *et al*. investigated in a randomized trial the effect of immediate extubation after coronary artery bypass graft (CABG) surgery compared to at least three hours ventilation before starting weaning on the pulmonary function. The study was performed in a PACU. They concluded that early extubation will not affect pulmonary function after extubation [16].

Our fast-track concept consists of direct postoperative treatment in a PACU with the primary goal of early extubation, followed by transfer to a step-down unit as soon as specific discharge criteria are met [6].

To the best of our knowledge, no prospective randomized study has been published which compares fast-track treatment in the ICU versus fast-track treatment in the PACU. The hypothesis of the study was that patients treated in the PACU would be extubated earlier, and be discharged to a step-down unit earlier than patients treated in the ICU. Accordingly, the objectives of our study were to compare ET and LOS in the PACU or ICU.

## Methods

The study was approved by our local ethics committee (Ethics Committee, Medical Faculty, University of Leipzig, Haertelstrasse 16–18, 04107 Leipzig, Reference number 097–2008, trial registration number ISRCTN71768341, http://www.controlled-trials.com/ISRCTN71768341/, registered 11 March 2014), and was conducted as a prospective, randomized, single-blinded, single-centre trial.

For each patient, written informed consent was obtained prior to any protocol-related activities. As part of this procedure, the principal investigator or designee explained orally and in writing the nature, duration, and purpose of the study in such a manner that the patient was aware of the potential risks, inconveniences, or adverse effects that may occur. The patient was informed that he/she was free to withdraw from the study at any time. The patient received all information that was required by local regulations and International Conference on Harmonisation (ICH) guidelines.

**Figure 1:**
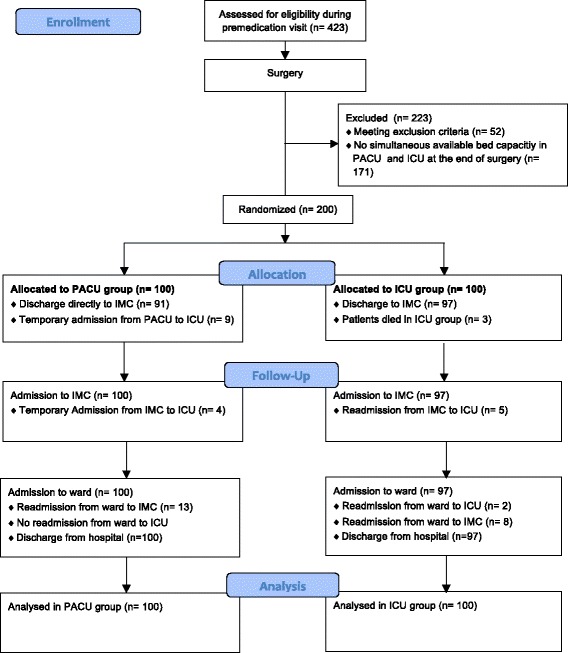
**Study flowchart.**

During the premedication visit the day before surgery, every patient scheduled to undergo CABG, valve surgery, or combined CABG and valve surgery was screened for inclusion in the study (Figure 1). Patients who were in cardiogenic shock, were dialysis dependent, or had an additive EuroSCORE of more than 10 were excluded.

The final decision for including or excluding the patient into the fast-track concept was taken by consensus decision between the attending anaesthesiologist and cardiac surgeon at the end of their surgery. Inclusion criteria were: haemodynamically stable (systolic blood pressure >90 mmHg and heart rate <120 bpm; adrenaline or noradrenaline <0.04 mcg/kg/min), normothermic (>36°C core body temperature), and no bleeding. Exclusion criteria followed risk factors identified by Constantinides *et al*. and Akhtar *et al*. [17,18]: impaired left ventricular function (ejection fraction below 35%), cardiac assist devices pre- or postoperative and cardiopulmonary instability postoperative (high inotropic support, lactate >5 mmol/l, Horowitz index below 200) After the decision to include the patient into the study, the patient was randomized to either postoperative care in the PACU (n = 100) or ICU (n = 100). For that purpose an envelope was picked out of a box containing 200 sealed envelopes (100 for PACU, 100 for ICU admission) and removed from the box subsequently. A further intra-operative exclusion criterion was lack of an available bed in either the PACU or ICU. In such cases, the patient was not randomized, but was sent to the unit with an available bed, and excluded from the study and further analysis. The medical and nursing staff in the ICU and PACU had been informed about the design and the conduct of the study but were not informed as to which patients were enrolled in the study.

Data collection and analysis was performed by an independent person who was not part of the anaesthetic, surgical or ICU team, and who was not blinded to treatment allocation.

### Fast-track anaesthesia protocol

Anaesthetic management consisted of oral premedication with clorazepate dipotassium (20 to 40 mg) the evening before and midazolam (3.75 to 7.5 mg) on the day of surgery. Anaesthesia was induced with fentanyl (0.2 mg) and propofol (1.5 to 2 mg/kg). A single dose of rocuronium (0.6 mg/kg) was used to facilitate intubation. Analgesia was maintained throughout the case with a continuous infusion of remifentanil (0.2 mcg/kg/min), and for hypnosis during the pre- and post-cardiopulmonary bypass (CBP) period sevoflurane (0.8 to 1.1 minimum alveolar concentration (MAC)) was administered whereas during CPB a continuous propofol infusion (3 mg/kg/h) was used. A recruitment manoeuvre was carried out prior to weaning from CPB in order to prevent atelectasis. An external convective warming system with an underbody blanket (Bairhugger™, Arizant Healthcare; Eden Prairie, MN, USA) was used after weaning from CPB to ensure a core temperature of at least 36°C was maintained. For early postoperative analgesia, 1 g paracetamol was administered intravenously to each patient before skin closure. In difference to other studies, we did not include all patients or selected fast-track patients only preoperatively. All patients received the fast-track anaesthesia in the OR. We carefully selected fast-track patients at the end of surgery following the criteria identified as risk factors for fast-track failure [1,17,18]. The final decision to continue the fast-track protocol postoperatively was taken after the end of surgery. As our primary end point was postoperative ventilation time, we defined fast-track failure as postoperative ventilation of more than six hours. That was decided due the literature research where it ranged between three and nine hours [19,20].

### Treatment in PACU

All patients were transferred to the PACU intubated, mechanically ventilated with a remifentanil infusion of 0.1 mcg/kg/min. Administration of hypnotic agents was discontinued in the OR.

Postoperative analgesia consisted of an bolus of piritramide (0.1 mg/kg) on discontinuation of the remifentanil infusion, followed by bolus doses as required in 2 to 4 mg aliquots, plus regular paracetamol (1 g every six hours) to achieve a pain score between 2 and 4 on an analogue pain scale from 0 to 10. Patients were extubated when they were conscious and obeyed commands, had stable spontaneous ventilation with pressure support of 10 to 12 cmH_2_O, positive end-expiratory pressure (PEEP) of 5 cmH_2_O, fraction of inspired oxygen (FiO_2_) of ≤0.4, were haemodynamically stable, not bleeding (≤100 ml/h), and with no significant electrocardiographic abnormalities.

All patients received non-invasive bi-level positive airway pressure ventilation via a face mask for one hour (Elisee 350™, Saime, Savigny-le-Temple, France), immediately after extubation. Initially non-invasive ventilation was commenced at a pressure support of 10 to 15 cm H_2_O and a PEEP of 5 cmH_2_O. The FiO_2_ was 0.4. During the period of non-invasive ventilation the pressure support was adapted to patients’ needs.

Criteria for discharge to the intermediate care unit (IMC) were that patients must be awake, cooperative, haemodynamically stable (without inotropes) and have both acceptable respiratory pattern and blood gas analysis (pO_2_ > 70 mmHg, pCO_2_ < 50 mmHg). Chest-X-ray and electrocardiogram were performed in all patients to exclude major pathology.

The physician-to-patient ratio and the nurse-to patient-ratio were 1:3. The PACU operated daily Monday to Friday from 10:00 to 18:30.

### Treatment in ICU

All patients arrived in the ICU intubated, mechanically ventilated with a remifentanil infusion of 0.1 μg/kg/min. Administration of hypnotic agents was discontinued in the OR.

Postoperative analgesia consisted of a bolus of piritramide (0.1 mg/kg) on discontinuation of the remifentanil infusion, followed by bolus doses as required in 2 to 4 mg aliquots, plus regular paracetamol (1 g every six hours). A pain scale was not used on a regular basis for assessing pain. The need for an analgesic medication was estimated by nurses. Extubation criteria were identical to those in the PACU. Non-invasive ventilation after extubation was not implemented routinely. Further treatment in the ICU was determined by the ICU physician according to German guidelines for intensive care treatment in cardiac surgery patients [21]. Criteria for suitability to transfer to IMC were identical to those in the PACU.

The physician-to patient-ratio was 1:12 and the nurse-to-patient ratio was 1:2.

Substantial differences in PACU and ICU treatment are listed in Table 1.

**Table 1:** **Substantial differences in PACU vs. ICU treatment**

	**PACU group**	**ICU group**
Physician-to-patient ratio	1:3	1:12
Nurse-to-patient ratio	1:3	1:2
Physicians specialisation	All anaesthesiologists	Diverse specialisations (for example cardiac surgeon)
Beds available	3 bed unit	21 bed unit
Opening time	Limited opening time	Unlimited opening, 24 hours
Patient population	Only elective cardiac surgery patients after pre- and postoperative evaluation of fast-track suitability that PACU staff can focus on	Mixed, as in the PACU but additionally patients in need of physicians’ attention due to multimorbidity and severe diseases (for example non-fast-track patients)
Analgesia regime	Strict regime as described in method section	Performed more liberally according to nurses estimation
	Pain scale for pain assessment.	
Timing of extubation	As soon as extubation criteria were met	According to physicians’ estimation under consideration of overall situation on the ICU presupposed that extubation criteria were met
Weaning protocol	Performed by physician	Mainly nurse-driven
	Good compliance to the protocol	Compliance to the weaning protocol depended on the actual workload
Stop of analgosedation	Remifentanil stopped at arrival (after paracetamol and piritramid were administered	Remifentanil stop according to disposition of the intensivist under consideration of overall situation on the ICU
Non-invasive ventilation	Performed routinely	Performed in only 4% of our population
Discharge to step-down unit.	Patient were discharged to step-down unit as soon as they met discharge criteria	Discharge to the step-down unit depended on need for ICU beds

PACU, post-anaesthetic care unit; ICU, intensive care unit.

### Outcomes

Primary end points were ET and PACU/ICU LOS. Secondary outcome measures were hospital LOS, overall length of intensive care treatment (total ICT LOS), in-house mortality, low cardiac output, new-onset cardiac arrhythmia, respiratory failure requiring prolonged ventilation or re-intubation and incidences of surgical re-exploration and renal failure.

PACU/ICU LOS is defined as LOS in the PACU or ICU from the end of surgery until discharge to another unit. Additionally, secondary PACU/ICU LOS includes readmissions from step-down units to ICU as well as additional ICU time after transfer from the PACU to ICU based on medical or organisational circumstances.

IMC LOS is defined as LOS in IMC until discharge to a general ward.

Primary ICT LOS is defined as overall length of intensive care treatment (ICT) in PACU/ICU + IMC.

Total ICT LOS is defined as overall length of ICT in the PACU + ICU + IMC including readmission to a unit of higher care grade than a general ward and transfer from the PACU to the ICU.

If patients were transferred from the PACU to the ICU in case of medical or organizational circumstances, they were still analysed as being in the PACU group, although additional ICU LOS was not calculated in PACU/ICU LOS but in secondary PACU/ICU LOS and total ICT LOS. PACU patients who had to stay past 18:30 were admitted to the ICU for further treatment and were evaluated as described above.

Low cardiac output was defined as central venous saturation of <65% with a haematocrit of >30%. Cardiac arrhythmia included atrial fibrillation and atrioventricular block. Acute renal failure was defined as an increase in postoperative serum creatinine of at least three times the preoperative value, or a serum creatinine >150 μmol/l. Stroke was defined as a new transient or permanent motor or sensory deficit of central origin or unexplained coma.

### Statistical analysis

Sample sizes were calculated on the basis of data from a previous retrospective study at our institution [6] using SPSS 16.0 (SPSS Inc, Chicago, IL, USA). Using this data, we estimated that ET in the ICU compared to ET time in the PACU would occur four hours later and that the standard deviation would be approximately 500 min. We calculated that 93 patients per group would be required to demonstrate a significant reduction in ET with a power of 90% at significance level of 5%. Accounting for drop-outs and incomplete data, we aimed to recruit 100 patients per group.

Comparisons between the two independent groups (ICU vs. PACU) were performed using the Mann–Whitney *U* test for continuous data, Mantel-Haenzsel test for categorically ordered data (for example New York Heart Association (NYHA) score) and Fisher’s exact test for binary data (for example adverse events). A threshold of 0.05 was considered as significant. All analyses were performed using SPSS 18.0. Continuous parameters were described by median and interquartile range. Categorical data are described by class-wise allocation numbers. Binary data are described as number of events.

The primary end point of this study was time to extubation. We have not adjusted for multiple testing, so other comparisons are considered explorative.

## Results

A total of 423 patients consented to participate in the study. All patients were scheduled for CABG, aortic valve replacement (AVR), mitral valve repair/replacement (MVR) or a combination of these procedures (Table 2). A total of 223 patients were excluded intraoperatively, due to a lack of capacity in either the ICU or PACU (n = 171), or because they were considered unsuitable for fast-track management at the end of their surgery, according to our criteria listed above (n = 52). A total of 200 patients were therefore included in the study from May 2008 until July 2009, 100 in each group. There were significantly more female patients in the PACU group (36 vs. 22, *P* = 0.04) (Table 3). Patients randomized to the PACU group had significantly shorter surgery time (170 min [145; 195] vs. 190 min [160; 230]; *P* <0.001) and anaesthesia time (255 min [235; 285] vs. 270 min [245; 313]; *P* = 0.02) than those in the ICU group (Table 3). Because of at most weak correlations with our primary outcomes, we decided not to adjust the analysis of primary outcomes for these imbalanced base-line variables (not shown). Cross-clamp (XCL) time (64 min [51; 79] vs. 66 min [51; 80]; *P* = 0.69) and total cardiopulmonary bypass time (100 min [75; 127] vs. 99 min [79; 122]; *P* = 0.91) were not significantly different between groups. The number and type of operations performed in both groups are listed in Table 2. There was no significant difference in type of surgery.

**Table 2:** **Operations performed**

**Type of surgery:**	**PACU group (n = 100)**	**ICU group (n = 100)**	***P*** **value**
AVR (n)	26	31	0.53
MVR (n)	33	27	0.44
CABG on-pump (n)	19	31	0.07
CABG off-pump (n)	22	11	0.06
Combined procedures (%)	4	9	0.25

Combined procedures are valve replacement/repair + CABG or combined repair/replacement of two valves. PACU, post-anaesthetic care unit; ICU, intensive care unit; AVR, aortic valve replacement; MVR, mitral valve replacement/repair; CABG, coronary artery bypass.

**Table 3:** **Demographic data (median and corresponding interquartile range)**

	**PACU group (n = 100)**	**ICU group (n = 100)**	***P*** **value**
Patients (n)	100	100	
Age (years)	65 [55; 72]	66 [57; 72]	0.61
Gender (male/female)	64/36	78/22	0.04
EuroSCORE (0–10 points)	2 [1; 4]	2 [1; 3]	0.64
Ejection fraction in %	63 [55; 66]	60 [51; 65]	0.16
NYHA (NYHA 1–4)	17/49/34/0	16/56/26/2	0.92
COPD (n)	8	10	0.81
Neurological deficit (n)	9	5	0.41
Peripheral vascular disease (n)	13	8	0.36
Diabetes mellitus (n)	25	31	0.43
Renal insufficiency (n)	6	14	0.06
Operative time (min)	170 [145; 195]	190 [160; 230]	<0.001
Anaesthesia time (min)	255 [235; 285]	270 [245; 313]	0.02
CPB time (min)	100 [75; 127]	99 [79; 122]	0.91
Cross-clamp time (min)	64 [51; 79]	66 [51; 80]	0.69

PACU, post anaesthetic care unit; ICU, intensive care unit; NYHA, New York Heart Association; COPD, chronic obstructive pulmonary disease; CPB, cardiopulmonary bypass.

### Time to extubation

**Figure 2:**
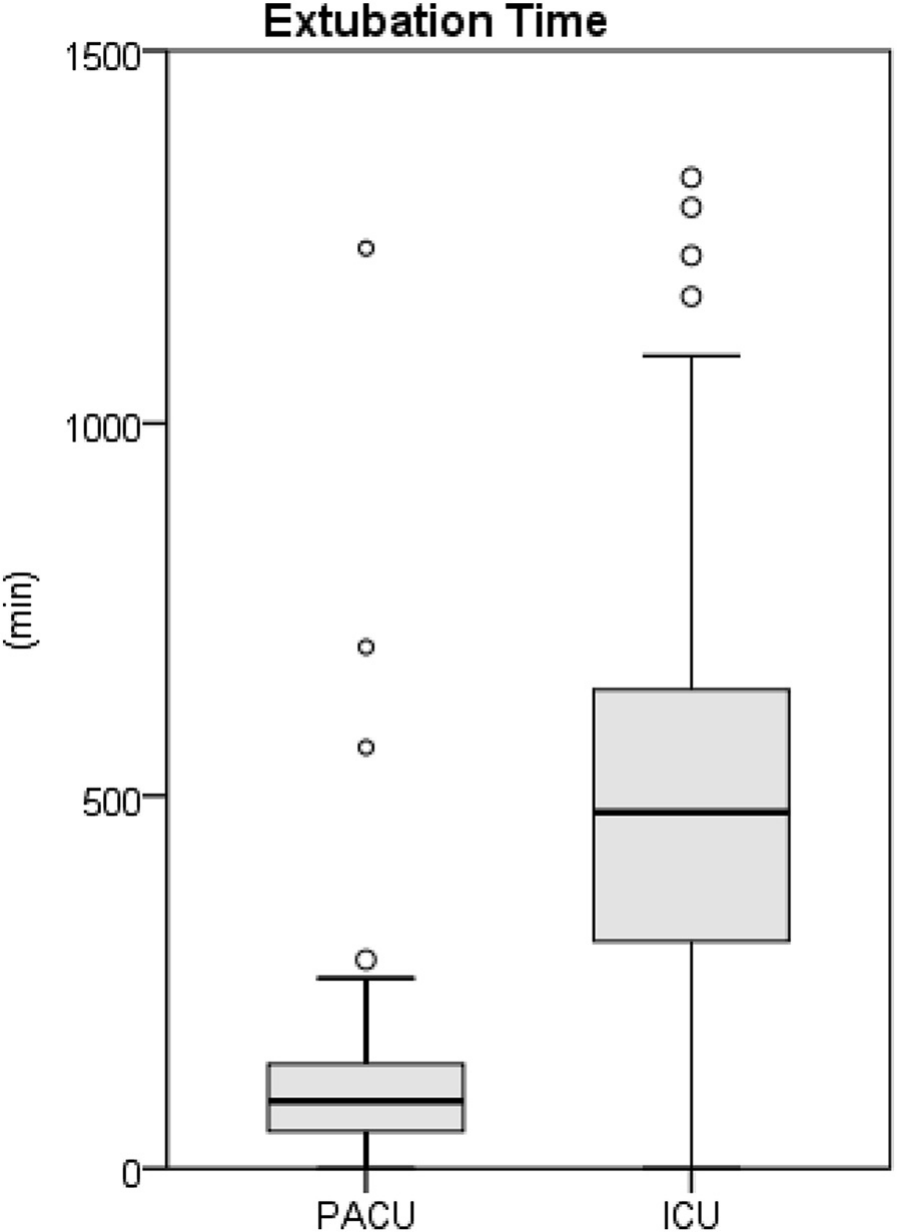
**Primary extubation time,** ***P*** **<0.001, outliers >1500 min are masked out (n = 3 in ICU group).**

The median extubation time in PACU group was significantly shorter than in the ICU group (90 min [50; 140] vs. 478 min [305; 643]; *P* <0.001; Figure 2, Table 4). In the PACU group 97% of the patients were extubated within six hours of admission whereas only 33% of the patients in the ICU group fulfilled the criteria for successful fast-tracking (*P* <0.001) [5].

**Table 4:** **Median extubation time and length of stay (LOS) and corresponding interquartile ranges**

	**PACU group (n = 100)**	**ICU group (n = 100)**	***P*** **value**
Primary extubation time (min)	90 [50; 140]	478 [305; 643]	<0.001
Extubation within 6 h (n)	97	33	<0.001
Reintubation (n)	5	10	0.28
Reintubation time (min)	930 [330; 1315]	990 [646; 6375]	0.68
Total ventilation time (incl. reintubation) (min)	105 [70; 175]	513 [320; 705]	<0.001
PACU/ICU LOS (hours)	3.3 [2.7; 4.0]	17.9 [10.3; 24.9]	<0.001
Readmission to ICU (n)	4	7	0.54
Secondary PACU/ICU LOS (hours)	3.5 [2.8; 5.1]	17.9 [10.3; 26]	<0.001
(incl. readmission from IMC to ICU and transfer from PACU to ICU)			
Secondary PACU/ICU LOS <24 hours (n)	95	71	<0.001
Primary IMC LOS (hours)	23.0 [19.9; 41.8]	21.0 [10.5; 28.8]	0.0035
Readmission IMC (n)	13	8	0.09
Primary ICT LOS (hours)	26.9 [23.2; 46.0]	41.1 [24.8; 60.2]	0.02
PACU + ICU + IMC excl. readmission			
Total ICT LOS (hours)	30.9 [23.9; 59.9]	43.9 [24.9; 65.4]	0.08
PACU + ICU + IMC incl. readmissions and transfer from PACU to ICU			
Hospital LOS (d)	9 [8; 11]	9 [8; 12]	0.42

Total ICT LOS = total intensive care treatment (ICU + IMC + PACU) length of stay including readmissions from step-down unit to unit of higher grade (reintubation time was calculated only for patients who were reintubated). PACU, post-anaesthetic care unit; ICU, intensive care unit; LOS, length of stay; IMC, intermediate care unit.

In the PACU group five patients required reintubation (three for resurgery, one because of a convulsion, and one for respiratory failure) compared to ten patients in ICU group (five for re-operation, four for respiratory failure, one for cardiopulmonary resuscitation). Additive ventilation time for reintubated patients was 930 min [330; 1315] in the PACU group vs. 990 min [646; 6375] in the ICU group (0.68).

Although the PACU had limited opening hours, time of arrival at the PACU seems not to have influence on ET.

### Length of stay in PACU, ICU, and hospital

**Figure 3:**
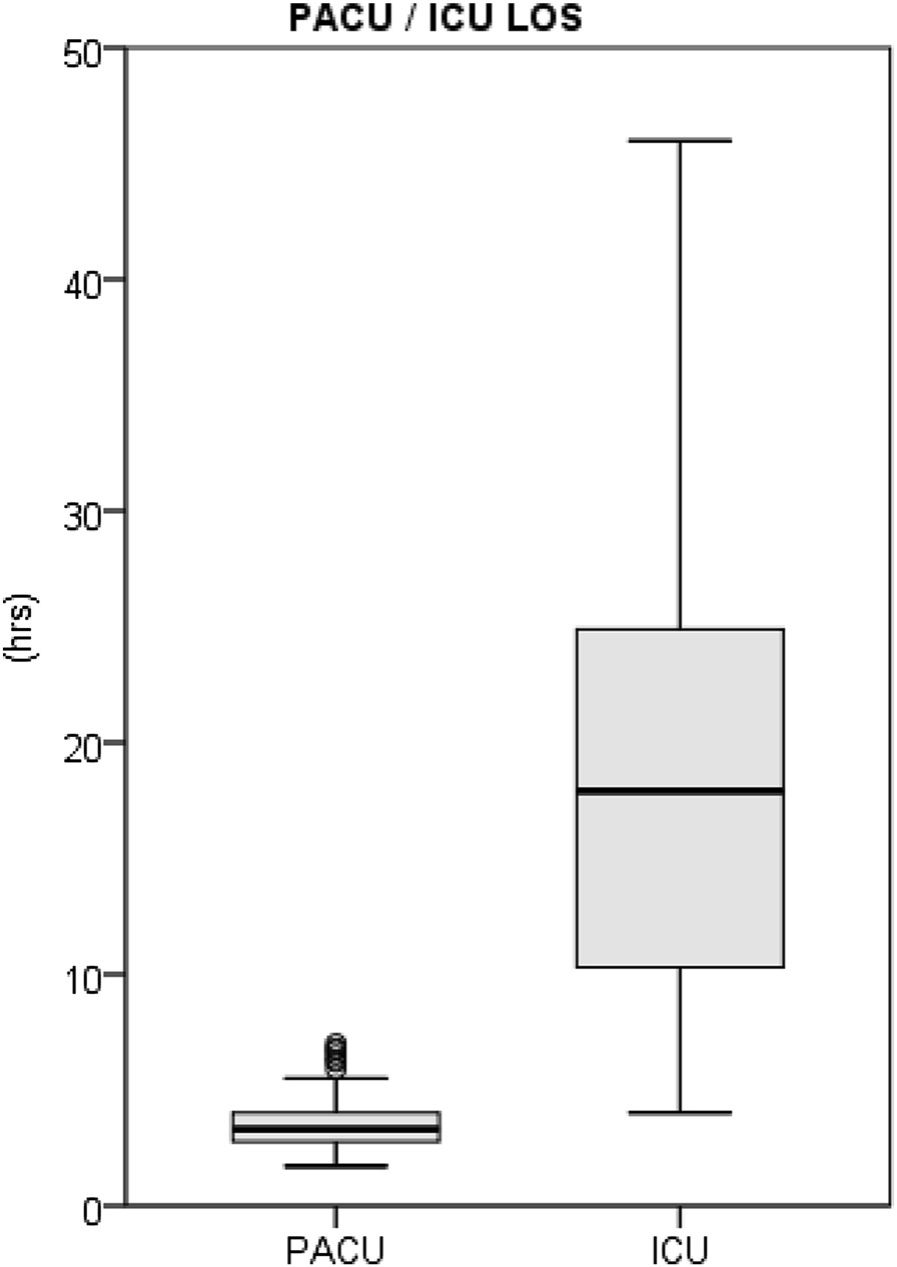
**Length of stay in post-anaesthetic care unit vs. intensive care unit,** ***P*** **<0.001, outliers >50 hours are masked out (n = 7 in ICU group).** ICU, intensive care unit.

The median LOS for the patients in the PACU group was 3.3 hours [2.7; 4.0] compared to 17.9 hours [10.3; 24.9] for patients in the ICU group (*P* <0.001; Figure 3, Table 4).

The median LOS in the IMC was 23.0 hours [19.9; 41.8] in the PACU group and 21.0 hours [10.5; 28.8] in the ICU group (*P* <0.004).

**Figure 4:**
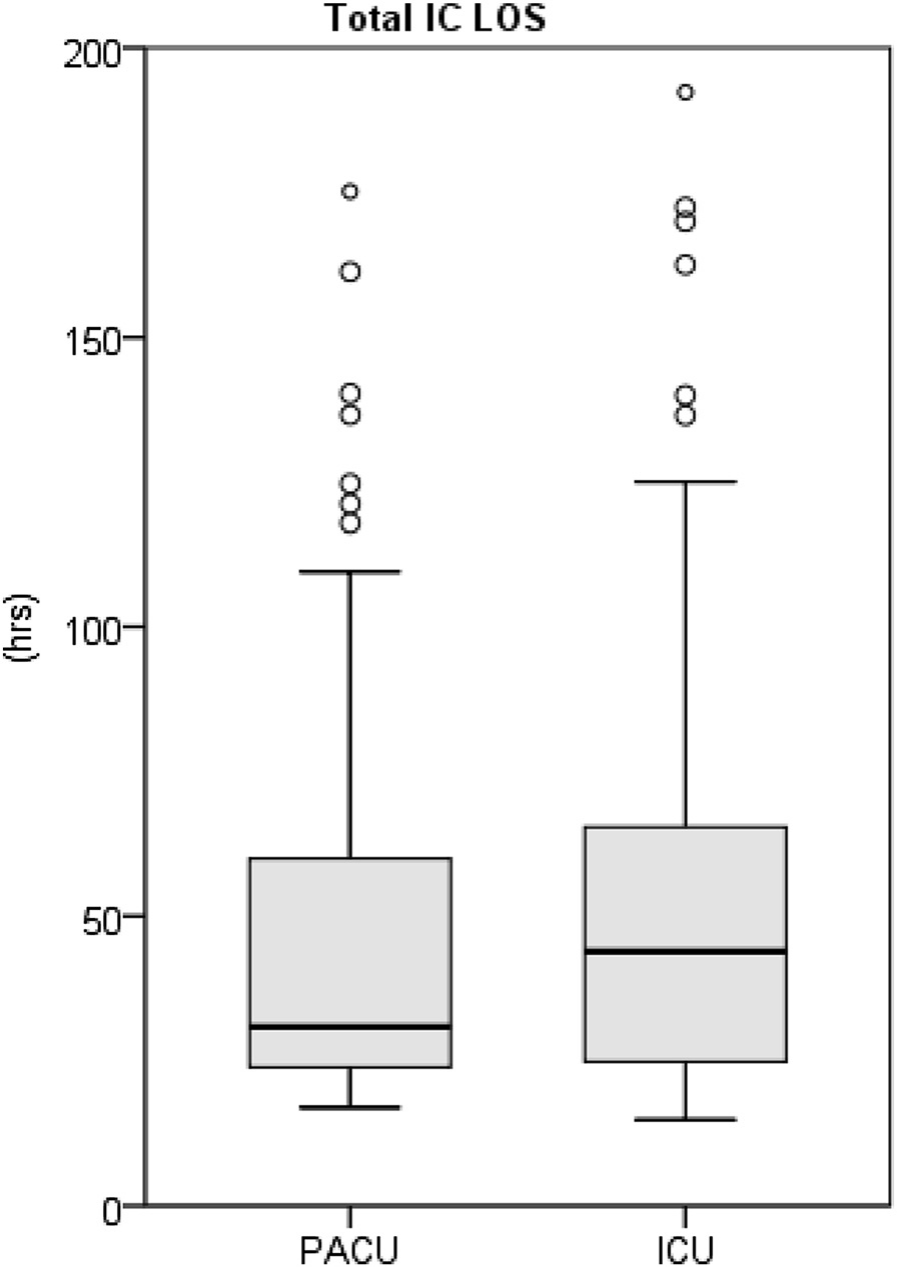
**Total ICT LOS = total intensive care treatment, length of stay in PACU/ICU + IMC including readmissions,** ***P*** **= 0.08, outliers >200 hours are masked out (n = 2 in PACU group, n = 6 in ICU group).** ICU, intensive care unit; IMC, intermediate care unit; LOS, length of stay; PACU, post-anaesthetic care unit.

Overall length of ICT in the PACU + ICU + IMC including readmission to a unit of higher care grade than a general ward and transfer from the PACU to the ICU was 30.9 hours [23.9; 59.9] for patients in the PACU group compared to 43.9 hours [24.9; 65.4] for patients in the ICU group (*P* = 0.08; Figure 4, Table 4).

There was no significant difference in median hospital LOS for the PACU group (9 [8; 11]) vs. the ICU group (9 [8; 12] days).

Ninety-one of 100 patients in PACU group were discharged to intermediate care unit whereas nine patients had to be admitted from the PACU to the ICU (Figure 1). Three of these were extubated and haemodynamically stable, and were admitted to the ICU because of lack of available beds in IMC, two patients because of failure to extubate, two patients because of bleeding, and two patients because of cardiac arrhythmia. Four patients in the PACU group had to be admitted from IMC to the ICU (two because of re-thoracotomy, one because of haemodynamic instability, and one because of respiratory failure). A total of 87% of all patients in the PACU group did not require any treatment in the ICU.

Readmission from the general ward to IMC occurred in 13 patients of the PACU group, and was due to: cardiac arrhythmia (n = 4), pleural effusion (n = 5), pneumothorax (n = 2), resurgery (n = 1), and pain control (n = 1), no patient in the PACU group discharged to the ward required readmission to ICU.

In the ICU group five patients required readmission from IMC to ICU, because of respiratory failure (n = 4) and cardiac arrest (n = 1). Two patients were readmitted from the general ward to ICU because they required resurgery. Furthermore, in the ICU group eight patients had to be readmitted from the general ward to IMC because of cardiac arrhythmia (n = 5), neurological deficit (n = 2) and pericardial effusion (n = 1).

### Postoperative complications

Postoperative complications for both groups are listed in Table 5. The occurrence of arrhythmias was significantly lower in the PACU group as compared to the ICU group (25 vs. 41, *P* = 0.02). There was no significant difference in the rate of pleural or pericardial effusions requiring intervention, renal insufficiency or cerebrovascular stroke.

**Table 5:** **Postoperative complications**

	**PACU group (n = 100)**	**ICU group (n = 100)**	***P*** **value**
Cardiac arrhythmia (n)	25	41	0.02
Unstable sternum (n)	1	1	1
Pleural or pericardial effusion (n)	22	14	0.20
Renal failure (n)	2	2	1
Reoperation (n)	5	11	0.19
Stroke (n)	0	2	0.50
Prolonged respiratory insufficiency >24 hours (n)	1	7	0.07
Low cardiac output (n)	0	3	0.25
Cardiopulmonary resuscitation (n)	0	5	0.06
Mortality (n)	0	3	0.25

PACU, post-anaesthetic care unit; ICU, intensive care unit.

The number of patients requiring resurgery (PACU n = 5 vs. ICU n = 11, *P* = 0.19) was lower in the PACU group (two for implantation of a pacemaker, two for drainage of a haemothorax, one for thrombectomy for deep vein thrombosis) compared to the ICU group (five for drainage of a haemothorax, two for revision of valve after valve replacement, two for implantation of a pacemaker, one thoracotomy for bleeding followed by insertion of extracorporeal membrane oxygenation after resurgery, one for refixation of the sternum).

One patient from the PACU group required ventilation longer than 24 hours vs. seven patients in ICU group (*P* = 0.07).

Compared to ICU patients none of the PACU patients developed low cardiac output syndrome (3 vs. 0, *P* = 0.25), needed cardiopulmonary resuscitation (5 vs. 0, *P* = 0.06), or died (3 vs. 0, *P* = 0.25), although there was no statistically significant difference.

## Discussion

In our study, we have shown that fast-track treatment of cardiac surgery patients in a dedicated PACU compared to fast-track treatment in the ICU significantly reduces ET (90 vs. 478 min; *P* <0.001) as well as time to transfer to a step-down unit (LOS PACU 3.3 hours compared to 17.9 hours LOS ICU). We were able to demonstrate a reduction of ventilation time and a significantly reduced utilisation of ICU capacity after cardiac surgery. Although we did not calculate the cost savings, Cheng *et al*. have clearly shown that early extubation results in reduced costs and better resource utilisation [4]. Hantschel *et al*. have also demonstrated that postoperative treatment in a PACU after cardiac surgery results in a 52% cost reduction compared to conventional ICU treatment [12]. Opening a PACU for 8.5 hours a day should lead to reduced personnel costs compared to a 24-hour ICU.

An ET of less than six hours after cardiac surgery is considered an important criterion for successful fast-tracking after cardiac surgery [4,5]. In the PACU group 97% of the patients fulfilled this criterion but only 33% in the ICU group (*P* <0.001). In a recent review, Zhu *et al*. showed that using a low-dose-opioid anaesthesia reduces ventilation times by 7.40 hours. Using a weaning protocol reduced ventilation times by 5.99 hours. In our study, we were able to reduce ventilation times by 6.46 hours, which is comparable to the reduction reported in other studies [11]. Our protocol used low-dose opioid anaesthesia with the short-acting opioid remifentanil. We defined a weaning protocol, which included early stop of anaesthesia, a protocol-driven postoperative pain management and non-invasive ventilation after extubation for at least 60 minutes.

Another fast-track criterion is reduced LOS in ICU, usually defined as less than 24 hours [5]. According to this criterion, successful fast-track-treatment was achieved in 95% of the PACU patients compared to 71% patients in the ICU group (*P* <0.001). Zhu *et al*. reported in a review a reduction in ICU LOS for low-dose-opioid anaesthesia of 3.7 hours (−6.98 to −0.41) and by using a weaning protocol of 5.15 hours (−8.71 to −1.59) compared to high-dose-opioid anaesthesia [11]. In our study, we achieved a reduction in PACU/ICU LOS by 14.6 hours to 3.3 hours. This early discharge to a step-down unit allows using an ICU bed more than once a day. Gooch *et al*. developed a model of demand elasticity of ICU bed utilization [22]. The authors discussed that ICU beds created their own demand [23]. Under the model of demand elasticity the case mix of patients in the ICU changed depending on bed availability. If enough beds are available or no actual patient needs an ICU bed, it is more likely that patients in the ICU who are not as critically ill do not benefit from ICU stay [23]. By bypassing the ICU for fast-track patients, we possibly reduced this effect of demand elasticity and were able to show a reduction in ICU bed utilization. Still, if we included the readmission and direct transfers from the PACU to the ICU, we found a significant reduction for ICU LOS of 14.4 hours (secondary ICU LOS PACU vs. ICU 3.5 to 17.9 hours).

Published figures for fast-track failure rates range from 11% to 49% depending on the patient population [17,18,24]. In contrast to studies that included all patients undergoing cardiac surgery, our study population was preselected according to our existing fast-track protocol. We primarily excluded patients with a defined risk for fast-track failure during the premedication visit (patients who were scheduled for emergency surgery, were in cardiogenic shock, were dialysis dependent, or had an additive EuroSCORE of more than 10) [1,17,25]. Another explanation for the low fast-track failure rate of 5% for the PACU group is the fact that the final decision for inclusion of the patient to fast-track treatment was made at the end of the surgery. Wong *et al*. identified need for inotropic support and bleeding as risk factors for delayed extubation as well as delayed LOS in ICU [26]. In our study, 52 out of the 423 patients primarily included were excluded before randomisation because of hemodynamic instability or bleeding at the end of the operation. This underlines the hypothesis that not only careful preselection of potential fast-track patients during the premedication visit is important, but also that re-evaluation of patient suitability at the end of the operation can lead to a reduction of fast-track failure. The relatively high fast-track failure rate for the ICU group (67% time to extubation >6 hours and 29% PACU/ICU LOS >24 hours) may be attributable to several factors: first, the much better physician-to-patient ratio in the PACU (1:3 in the PACU vs. 1:12 in the ICU) allows the physician to effectively implement and manage an early goal-directed therapy. Since the study from Rivers *et al*. in septic patients we know that early hemodynamic stabilisation is beneficial for the patient and this is certainly also true for cardiac fast-track patients [27]. Several other studies have shown that an early goal-directed fluid management in postoperative cardiac surgery patients results in an improved hemodynamic stability and can reduce ventilation time and ICU LOS [28,29]. Second, due to the fact that one physician in the ICU cares for 12 patients the preselected fast-track patients will not get the same attention as the patient who really needs ICT. One to two severely compromised patients out of the 12 will result in the fact that weaning of the fast-track patient on ICU will be delayed. Kumar *et al*. have shown that the presence of an intensivist results in reduced ETs [30]. Third, the limited opening times for the PACU may positively motivate the involved staff to treat the patients optimally including early extubation and hemodynamic and respiratory stabilisation so that the patient can be transferred to the IMC for further treatment.

Also, the more focused adherence to the fast-track and enhanced-recovery principles including specifications for medication, postoperative pain control and discharge criteria favours the PACU compared to the ICU. van Mastrigt *et al*. showed in a meta-analysis that a defined weaning-and-extubation protocol is an important key to reduced intensive care LOS [10]. Although this protocol was the same for the PACU and the ICU, the more disciplined execution of the fast-track protocol and application of non-invasive ventilation in our PACU might be another important factor for success of early extubation. In a prospective randomized study, Zarbock *et al*. demonstrated a significant reduction in reintubation and readmission to ICU/IMC in cardiac surgery patients using continuous positive airway pressure therapy [31].

We found a lower incidence of reintubation in the PACU with 2.5% (five) vs. 5% in the ICU (ten) patients and a lower readmission rate of the PACU (four) vs. the ICU (seven) patients from step-down unit (IMC) to the ICU without reaching significance. Zhu *et al*. reported a risk of reintubation in the fast-track group of 1.4% and in the conventional group of 1.7%, [11], which is lower as in our study. However, this study is underpowered to allow any conclusion to the reintubation rate compared to other studies.

The incidences of low cardiac output syndrome, prolonged respiratory insufficiency, cardiac arrest, and death tended to be lower in the PACU group without reaching statistical significance. Because these complications were not primary end points, our study was underpowered for demonstrating significant differences between groups. The incidence of renal failure, stroke, resurgery, and mortality was similar for the PACU and the ICU group. Our study does not allow any conclusion about the safety of our fast-track concept. However, a significantly lower incidence of common postoperative complications for fast-track patients was demonstrated in a prospective study of 1,488 patients by Gooi *et al*. [3]. Svircevic *et al*. could not find any evidence for increased risk of adverse outcomes in 7,989 patients undergoing fast-track cardiac surgery [5]. In a recent review, Zhu *et al*. came to the conclusion that fast-track interventions have similar risks of mortality and major postoperative complications to conventional (not fast-track) care, and therefore appear to be safe in patients considered to be at low to moderate risk [11].

In contrast to other studies on fast-track in cardiac surgery, which included only patients undergoing coronary artery bypass surgery, our patient population was mixed regarding type of operations [4,10,19,32]. More than half of our patient population underwent valve surgery, some of them in combination with CABG. Overall, in our patient population of n = 200 patients only 41.5% were CABG patients (41 vs. 42). A total of 6.5% of all patients (four vs. nine) underwent combined procedures (for example aortic and mitral valve surgery or valve surgery and CABG). We have also shown that fast-track treatment utilising a dedicated PACU can be successfully implemented for different types of cardiac operations.

### Limitations of the study

Our demographic data show that there is a significant difference in gender (more female patients in the PACU group). In several studies, female gender was found to be a risk factor for delayed postoperative extubation and prolonged ICU length of stay [1,26]. This might have favoured the ICU group. Anaesthesia and surgery time in the ICU group was significantly longer, but there was no difference in XCL and cardiopulmonary bypass time, which were (amongst others) identified as risk factors for delayed postoperative extubation (>6 hours) and prolonged ICU LOS (>24 hours) [1,26]. Regarding anaesthesia and surgery time, we observed only weak correlations with our outcome variables in both PACU and ICU groups. Hence, it is unlikely that this imbalance in baseline characteristics affects the main conclusion of our study.

Regarding the adverse events, the study was not adequately powered to identify significant differences between the groups.

## Conclusions

Our study showed that our fast-track treatment in a dedicated PACU leads to a high rate of success (95%) compared to the ICU (33%). We attribute this difference to better physician-to-patient ratio, allowing for more focused, early postoperative management, and better adherence to an established fast-track protocol. Delaying the decision about patient suitability for fast-track treatment until the end of surgery may also contribute to reducing the incidence of fast-track failures. Running a PACU separated from the ICU in a different part of the hospital, an excellent physician-patient ratio and strong adherence to the fast-track protocol is from our point of view one of the success factors for our study.

## Key messages

- ET for cardiac surgery patients in a fast-track protocol is significantly shorter in a dedicated PACU than in ICU
- PACU-LOS is significantly shorter than ICU-LOS

## Abbreviations

AVR: aortic valve replacement
CABG: coronary artery bypass graft
CBP: cardiopulmonary bypass
COPD: chronic obstructive pulmonary disease
ET: time to extubation
FiO_2_: fraction of inspired oxygen
ICT: intensive care treatment
ICU: intensive care unit
IMC: intermediate care unit
LOS: length of stay
MAC: minimum alveolar concentration
MVR: mitral valve replacement/repair
NYHA: New York Heart Association
OR: operating room
PACU: post-anaesthetic care unit
PEEP: positive end-expiratory pressure
XCL: cross-clamp
